# Clinicopathological features of mammary Paget’s disease: a single-center experience in Turkey

**DOI:** 10.3906/sag-2103-148

**Published:** 2021-09-07

**Authors:** Haldun KAR, Sultan Deniz ALTINDAĞ, Demet ETİT, Seyran YİĞİT, Nihan ACAR, Mustafa Agah TEKİNDAL, Özlem GÜR, Betül KÜÇÜKZEYBEK, Özgün AKGÜL, Kemal ATAHAN

**Affiliations:** 1 Department of General Surgery, Katip Çelebi University Atatürk Training and Research Hospital, İzmir, Turkey; 2 Department of Pathology, Nevşehir State Hospital, Nevşehir, Turkey; 3 Department of Pathology, Katip Çelebi University Atatürk Training and Research Hospital, İzmir, Turkey; 4 Department of Biostatistics, Katip Çelebi University Faculty of Medicine, İzmir, Turkey; 5 Department of General Surgery, Ege Şehir Hospital, İzmir, Turkey

**Keywords:** Paget’s disease, breast carcinoma, pathology, molecular subtype, immunohistochemistry

## Abstract

**Background/aim:**

Paget’s disease (PD) of the breast is a very rare presentation of breast malignancy, accounting for 1%–3% of all primary breast tumors. We aimed to evaluate and compare the clinicopathological features and clinical outcome of PD accompanied by in situ carcinoma and invasive cancer.

**Materials and methods:**

We used the archive of our pathology laboratory retrospectively for age, sex, history of surgery, histopathological findings, treatment modalities, and follow-up information. We used the Kaplan–Meier method for survival analysis.

**Results:**

There were 46 female patients diagnosed with PD. In 39 (84.7%) patients, invasive carcinoma accompanied PD, while 7 (15.3%) patients had ductal carcinoma in situ. The median age at diagnosis was 53.5 years. The median follow-up period was 47 months. Of the 39 invasive carcinoma, 10 (25.6%) died during the follow-up period. Invasive ductal carcinoma group had a mean overall survival of rate of 57.8 ± 6.6 months. According to univariate analysis, only the tumor type was found to impact overall survival (p < 0.001).

**Conclusions:**

The current study displayed the tumor type as the only parameter affecting overall survival in the invasive carcinoma group. Although it was not statistically significant, breast cancers accompanied by PD were found to be predominantly advanced stage tumors, high grade, hormone receptor negative, and HER2 positive.

## 1. Introduction

Paget’s disease (PD) of the breast is a very rare presentation of breast malignancy, accounting for 1%–3% of all primary breast tumors [1–3]. PD of the breast emerges as erythematous and ulcerated nipple. As these changes are frequently diagnosed as dermatitis or eczema, the diagnosis and treatment are often delayed. The diagnosis must be confirmed with a biopsy of the nipple-areola complex [4–6]. It is characterized histopathologically by the infiltration of the nipple epidermis with relatively large round tumor cells with clear cytoplasm and vesicular nuclei with hyperchromatic nuclei [7]. There are two theories on the hypothesis of the nature and origin of PD: in situ malignant transformation theory and the epidermotropic theory. The transformation theory tells us that Paget cells are transformed from keratinocytes of the epidermis of the nipple. The second theory assumes that Paget cells are ductal carcinoma cells that have migrated from the underlying breast ducts to the epidermis of the mammary gland. Regardless of the origin the Paget cells, this is still debated [4,8,9]. Most cases have an underlying in situ or invasive breast carcinoma and some cases appear without any underlying neoplasia [4,5,10]. In different series, while ductal carcinoma in situ (DCIS) associated with Paget’s disease is frequently comedo type, invasive ductal carcinoma is the most common type of invasive cancer associated with PD [4,8]. Coexistence of PD with invasive cancer was found to be associated with poor prognosis, and various clinicopathological parameters were implicated in the studies conducted to reveal the reason for this [8,10].

In recent years, in line with the advances in diagnosis and treatment of breast cancer, there have been significant developments in pathological diagnostic criteria of PD and its surgical approach. Considering these developments, we aimed to evaluate and compare the clinicopathologic characteristics and clinical outcome of PD accompanied by in situ carcinoma and invasive cancer.

## 2. Materials and methods

### 2.1. Case selection

We searched the archive of our pathology laboratory retrospectively and we collected the clinical information from our hospital’s electronic database. We selected 54 patients diagnosed with PD between December 2006 and June 2017. We excluded 3 patients whose data were not available and 5 patients who had metastasis. We included 46 patients in our study. A detailed histopathologic review was performed by three pathologists to confirm the diagnosis of PD. While the diagnosis of PD in 37 patients was based on the skin biopsy and/or mastectomy material performed in our hospital, the diagnosis of 9 patients was made in consultation blocks. We contacted the patients diagnosed by consultation blocks and we obtained the necessary information from the patients themselves and/or their relatives. We obtained information such as age, sex, history of surgery, histopathologic findings, treatment modalities, and follow-up information. The cases were divided into two groups as PD-DCIS and PD-Invasive carcinoma (IC) according to the pathology accompanying Paget’s disease. Age, laterality, tumor location, tumor size, tumor type and type of surgery were regarded as clinical characteristics, while tumor grade, ER, PR, HER2 status, Ki-67, molecular subtype and lymph node status were regarded as histopathological characteristics; their effect on overall survival (OS) was investigated.

The study protocol was approved by the institutional ethics committee of Izmir Katip Celebi University (22.10.2020-1008).

### 2.2. Histopathological evaluation

During histopathological examination, presence of underlying tumor, tumor diameter, tumor type, in situ carcinoma type, presence of lymph node metastasis, histological type of tumor and tumor multifocality were recorded. Immunohistochemical stainings were reevaluated. All antibodies were provided from Dako Cytomation (Denmark) and they were ready to use. In order to determine the antibody distribution pattern, percentage of positive cells and intensity of reactive tumor cells were scored semiquantitatively for estrogen receptor (ER) and progesteron receptor (PR). A positive result was considered if at least 1% of cells have a nuclear expression [11]. Human epidermal growth factor receptor 2 (HER2) was scored using the new recommendations of ASCO/CAP Guidelines. Cases with immunohistochemically 2 + score were further analyzed for HER2 gene amplification by fluorescence in situ hybridization technique [12]. Less than 14% positive nuclear staining for Ki-67 antibody was considered low expression [13]. p53 was considered positive if more than 10% of tumor nuclei were stained. In terms of immunohistochemical expressions of ER, PR, HER2 and Ki-67, the tumor was classified into following molecular subtypes: Luminal A (ER+ and/or PR+, HER2−, low Ki-67), Luminal B (ER+ and/or PR+, HER2+, any Ki-67 or ER+ and/or PR+, HER2−, high Ki-67), HER2 rich (ER−, PR−, HER2+), triple negative (ER−, PR−, HER2−) [14].

For tumor location, physical examination and gross pathology assessment were evaluated together. Invasive tumors were graded according to the modified Scarff-Bloom-Richardson grading system. For in situ carcinomas, nuclear grading was noted as mild, moderate, or severe. Comedo necrosis was also recorded in all cases. The staging was performed according to American Joint Committee on Cancer 8th edition [14].

### 2.3. Statistical analysis

Survival analysis was done by using the Kaplan–Meier method. Comparison of the variables of the survival times of the factors between the categories was evaluated by the log-rank Mantel–Cox test. OS was defined as the duration from initial diagnosis to death due to any cases. The data were evaluated via SPPS 20 (IBM Corp. Released 2011. IBM SPSS Statistics for Windows, Version 20.0. Armonk, NY: IBM Corp.). p < 0.05 and p < 0.01 were taken as significance levels.

## 3. Results

There were 46 patients diagnosed with PD, all were female. Invasive carcinoma accompanied PD in 39 (84.7%) patients, while DCIS was detected in 7 (15.3%) patients. Most of the invasive tumor type was invasive ductal carcinoma (IDC) (76.9%), eight patients with mixed type had invasive ductal cancer accompanied by invasive papillary, invasive lobular, invasive micropapillary and glycogen-rich type. One patient (2.6%) was with pure apocrine features. All in situ carcinomas were ductal carcinoma in situ. The median age at diagnosis was 53.5 (range: 30–80) years, which was 45.4 and 54.9 in DCIS group and IC group, respectively. Most of the patients had unifocal tumor either at central or peripheral location. Locations of IC and DCIS are shown in Table 1. While the diagnosis was made by skin biopsies taken from the areola in 11 patients, mastectomy specimen displayed the diagnosis in 35 patients.

In terms of surgical techniques, modified radical mastectomy (MRM), breast conserving surgery (BCS), simple mastectomy, and nipple areola complex (NAC) resection were performed in 32, 7, 6, and 1 patient, respectively. Modified radical mastectomy in the IC group and BCS in the DCIS group were the preferred surgical methods. Sentinel lymph node biopsy (SLNB) was performed in 5 of 39 patients diagnosed with IC, and axillary lymph node dissection (ALND) was performed in 32 patients. While SLNB was negative in 4 patients, metastatic lymph node was detected in one patient and a subsequent ALND was performed. Three of 32 patients who underwent ALND did not have metastases and the remaining 29 patients had metastatic lymph nodes. While SLNB was performed in 4 of 7 patients with DCIS, ALND was performed in 2 patients, and axillary sampling was not performed in one patient. Lymph node metastasis was not detected in this group. Surgical procedures for all patients, except for one patient who refused the surgical treatment after biopsy, are summarized in Table 1.

The data of histologic tumor diameter was available in 36 of 39 invasive carcinomas, and the mean tumor diameter was 3.4 (0.1–12) cm. The mean tumor diameter in DCIS group was 1.025 (0.5–1.5) cm.

In our study, hormone receptor levels were low and HER2 levels were high in the PD-IC group. In the molecular subtype analyses, HER2 rich group was the highest. In terms of molecular subtypes among the deceased 10 patients, 2 patients were luminal A, 4 patients were luminal B HER2 +, 3 patients were HER2 rich, and one patient was triple negative. Six patients from DCIS group had high nuclear grade, while only one case had low nuclear grade. Comedo necrosis was detected in 3 of these cases, and one case was noncomedo DCIS. All pathological features are summarized in Table 2. None of the parameters mentioned above was statistically significant when the two groups were compared.

While distant metastasis occurred in two patients from PD-IC group during the first and second years of their follow-up, one patient from PD-DCIS group (external consultation) developed metastatic lymph nodes with perinodal invasion in the ipsilateral axilla 3 years after the index operation (mastectomy + SLNB).

**Figure 1:**
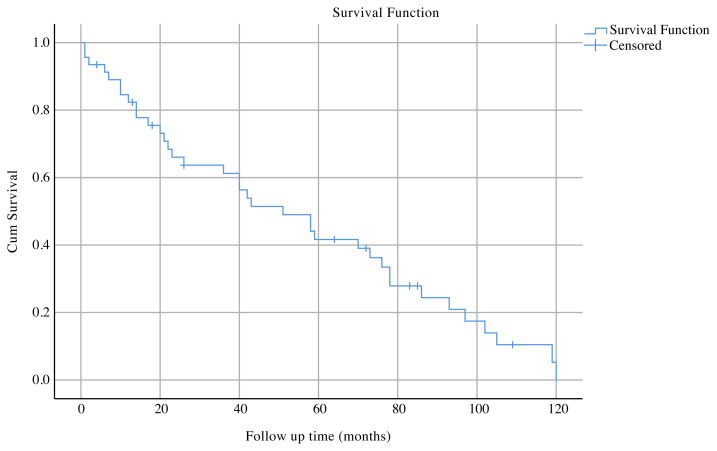
Kaplan–Meier curves for cumulative survival of the IC group.

**Figure 2:**
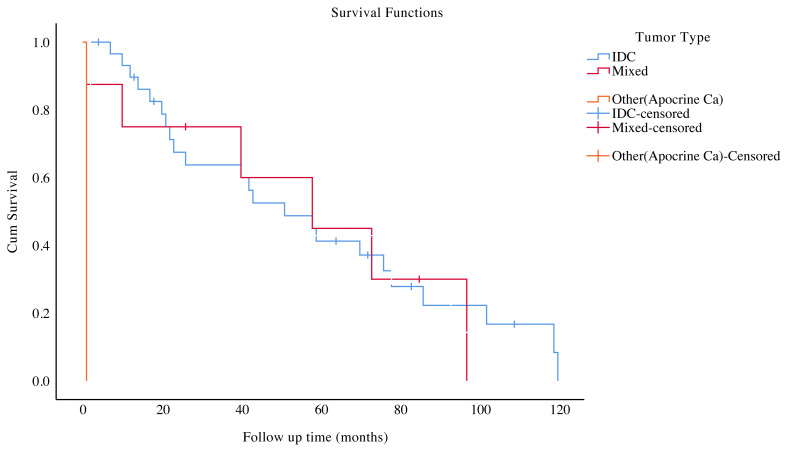
Kaplan–Meier survival plots according to tumor type.

The median follow-up period was 47 months (range: 1–120 months). Of 39 IC patients, 10 (25.6%) died during the follow-up period. Among the 7 patients with the available data of the cause of death, 5 had metastatic disease, one developed pneumonia during chemotherapy, and one had a myocardial infarction. Mortality was not observed in PD-DCIS group. Invasive carcinoma group had a mean OS of 57.8 ± 6.6 months (95% CI: 44.8–70.8) and median OS of 58 ± 9.5 months (95% CI: 39.2–76.7) (Figure 1). According to univariate analysis, only the tumor type was found to impact OS (p < 0.001) (Figure 2). Invasive ductal carcinoma had poor OS when compared with mixed type. Other clinicopathologic variables were not indicators of prognosis.

## 4. Discussion

Paget’s disease is a rare condition with gradually decreasing incidence [15]. Studies on PD are mostly the single-center cohort or case series, except for SEER database studies; therefore, results vary accordingly. On the other hand, it was shown that invasive cancers accompanied by PD had a more aggressive pathological character and had a worse prognosis than invasive breast cancers without PD in most studies [8,10,16,17].

Paget’s disease is frequently encountered in females, in the 5th–6th decades of life [3,8,10,18]. In our study, the mean age was 53.5 years. In addition, patients from the DCIS group were younger than those in the IC group.

Although Helme et al., in their review, reported that multifocality ranged between 21% and 80% in mastectomy specimens, recent studies have shown that multifocality and/or multicentricity rates are below 40% [2,19,20]. In our study, this rate was found to be 30.8% in the IC group and 28.6% in the DCIS group. Some studies also have reported that NAC localization of the tumor is more common in the DCIS group than in the IC group [10,15]. Our study also supported this data since NAC localization was detected 57.1% and 25.6% of the patients with DCIS and IC, respectively.

In the past, mastectomy was accepted as the standard surgical procedure with the view that patients diagnosed with Paget’s disease may have multifocal/multicentric disease or occult tumor foci located outside the nipple in the breast tissue [10]. This approach has recently changed, and BCS + RT is accepted as an effective local treatment method in selected cases [2,3,5]. Additionally, SLNB is also recommended instead of routine axillary dissection in selected cases [3,15]. In our study, mastectomy (92.3%) in the IC group and breast-conserving surgery (72.4%) in the DCIS group were the most preferred methods. SLNB was also performed in most of the cases with DCIS (71.5%). All patients, who underwent BCS, NAC excision, and biopsy, received adjuvant RT.

More than 90% of PD is associated with invasive cancer and the most common type of invasive cancer is invasive ductal cancer. Invasive lobular cancer was reported only in four studies in the literature, and the other specific types of breast cancer accounted for less than 10% [2]. In our study, 84.7% of the cases were invasive carcinoma, and among them, 76.9% were invasive ductal carcinoma. In univariate analysis, tumor type was the only significant parameter affecting the OS. Like the previous studies, IDC was found to be associated with poor prognosis [6,18,22]. Müjgan et al. found that cancer-specific survival was significantly worse for patients with invasive disease [4].

Wachter et al. emphasized that PD accompanying low-grade DCIS was uncommon, while Chen et al. found that some of DCIS cases accompanied by PD were associated with high mortality by displaying invasive cancer behavior [10,21]. In a study by Wong et al. [15], lymph node metastases were detected in 4.1% of DCIS patients which was due to an undetectable occult invasive cancer. In our study, six of seven patients with DCIS had high nuclear grade, and axillary lymph node metastasis was detected in one patient (14.2%) during follow-up.

Grade 2 and 3 tumors have been detected in 83.4%–100% of PD-IC cases [3,4,6,8–10,23]. This rate was 92.4% in our study, which was consistent with the literature.

Studies have shown that in invasive breast cancers accompanied by PD, hormone receptor positivity is lower and HER2 positivity is 2 to 4 times higher than invasive cancers without PD [2,4,9,10]. Although low hormone receptor (HR) positivity and high HER2 positivity have been shown to be effective on prognosis in many studies, there is also exceptional study showing that these factors do not have any impact on prognosis [10]. ER, PR, and HER2 were detected as positive in 28.2%, 35.9%, and 64.1% of our cases in accordance with the literature, but their effects on prognosis were not statistically determined.

Molecular subtype analysis has an important effect on the choice of treatment and determining the prognosis of the disease. HER2 rich and triple negative groups demonstrate aggressive clinical behavior. When the literature on molecular subtypes indicate that HER2 rich and Luminal B subgroups are predominantly higher in patients with PD-IC (Table3) [21,24–26]. In our study, most invasive cancers (79.5%) with the available data of immune markers were HER2 positive (64.5%), and most of them were hormone receptor negative (41.9%). 40% of the metastatic cases and 42.8% of the patients who died during the follow-up were in the HER2 rich group.

High rates of lymph node involvement, between 48% and 69%, in PD accompanied by invasive cancer have been reported [2]. The study of Wong et al. [9] has shown that the presence of PD is a statistically significant marker for axillary metastasis in invasive breast cancer. The higher rate (76.9%) of lymph node metastasis in our study compared to previous studies was attributed to the fact that more than half of the patients in the IC group had advanced stage tumor at first admission.

In conclusion, there was no statistically significant difference in terms of clinicopathological features when PD-DCIS and PD-IC were compared. In addition, the current study displayed the tumor type as the only parameter affecting OS in the IC group. On the other hand, although it was not statistically significant, breast cancers accompanied by PD were found to be predominantly high grade and/or advanced stage tumors, HR negative and HER2 positive. In our study, HER2 rich subtype was the most frequently observed molecular subtype. In addition, considering that we may encounter PD-DCIS cases more frequently in the near future, a close follow-up in PD cases accompanied by DCIS would be beneficial to prevent locoregional recurrence.

## Figures and Tables

**Table 1:** Clinical characteristics of the patients.

Characteristic	PD-DCIS n = 7 (%)	PD-IC n = 39 (%)
**Age (years)**		
<40	2 (28.6)	7 (18)
40–59	5 (71.4)	15 (38.4)
≥60	0	17 (43.6)
**Laterality**		
Left	4 (57.1)	22 (56.4)
Right	3 (42.9)	17 (43.6)
**Tumor location**		
Central	4 (57.1)	10 (25.6)
Unifocal peripheral	0	12 (30.8)
Multicentric	2 (28.6)	12 (30.8)
Unknown	1 (14.3)	5 (12.8)
**Type of surgery**		
MRM	0	32 (82)
BCS + SLNB	3 (42.9)	2 (5.1)
BCS + ALND	1 (14.3)	0
Simple mastectomy +SLNB	2 (28.5)	3 (7.7)
Sımple mastectomy	0	1 (2.5)
NAC resection	1 (14.3)	0
NAC biopsy	0	1 (2.5)
**Tumor size**		
0–2 cm	5 (71.4)	15 (38.4)
2.1–5 cm	1(14.3)	14 (35.9)
>5 cm	0	7 (18)
Unknown	1 (14.3)	3 (7.7)
**Tumor type**		
Invasive ductal carcinoma	-	30 (76.9)
Mixed tumor	-	8 (20.5)
Apocrine tumor	-	1 (2.6)
Ductal carcinoma in situ	7 (100)	-

PD-DCIS = Paget’s disease-ductal carcinoma in situ; PD-IC = Paget’s disease-invasive carcinoma; MRM = modified radical mastectomy; BCS = breast-conserving surgery; SLNB = sentinel lymph node biopsy; ALNB = axillary lymph node dissection; NAC = nipple areola complex.

**Table 2:** Histopathologic characteristics of the tumors.

Characteristic	PD-DCIS n = 7 (%)	PD- n = 39 (%)
**Tumor Grade**		
Grade 1	-	2 (5.1)
Grade 2	-	21 (53.9)
Grade 3	-	15 (38.5)
Unknown	-	1 (2.5)
**ER status**		
Positive	2 (28.6)	11 (28.2)
Negative	5 (71.4)	28 (71.8)
**PR status**		
Positive	0	14 (35.9)
Negative	7 (100)	25 (64.1)
**HER2 status**		
Positive	5 (71.4)	25 (64.1)
Negative	1 (14.3)	13 (33.4)
Unknown	1 (14.3)	1 (2.5)
**Ki-67 (%)**		
≤15	-	9 (23.1)
>15	-	22 (56.4)
Unknown	-	8 (20.5)
**Molecular subtype**		
Luminal A	-	2 (5.1)
Luminal B HER 2 +	-	7 (18)
Luminal B HER 2 −	-	4 (10.2)
HER 2 rich	-	13 (33.4)
Triple negative	-	5 (12.8)
Unknown	-	8 (20.5)
**Lymphnode status**		
pN0	6 (85.7)	8 (20.5)
pN1	0	12 (30.8)
pN2	0	10 (25.6)
pN3	0	8 (20.5)
Unknown	1 (14.3)	1 (2.6)

PD-DCIS = Paget’s disease-ductal carcinoma in situ; PD-IC = Paget’s disease-invasive carcinoma; ER = estrogen receptor; PR = progesterone receptor; HER2 = human epidermal growth factor receptor 2.

**Table 3:** Molecular subtype analyses in PD-IC in the literature.

	Molecular subtype, n (%)				
	HR+/Her2 −	HR+/Her2 +	HR−/Her2 +	HR−/Her2 −	Unkown
Chen et al.^10^	121 (30.5)	132 (33.2)	119 (30.0)	25 (6.3)	-
Lee et al.^1^	3 (9.4)	4 (12.5)	22 (68.8)	3 (9.4)	-
Wu et al.^3^	139 (19.1)	129 (27)	115 (24.1)	28 (5.9)	66 (13.8)
Yao et al.^5^	108 (3.5)	114 (3.7)	107 (3.5)	23 (0.8)	2695 (88.4)
Wong et al.^9^	40 (28)	47 (32.9)	49 (34.2)	7 (4.9)	-
Arafah et al.^24^	2 (10)	4 (20)	9 (45)	5 (25)	-
Wahcter et al.^21^	-	10 (50)	8 (40)	2 (10)	-
Sek et al.^25^	1 (8)	1 (8)	10 (84)	-	-
Lester et al.^26^	-	3 (30)	4 (40)	3 (30)	-
Present study	6 (15.3)	7 (18)	13 (33.4)	5(12.8)	8 (20.5)

PD-IC = Paget’s disease–invasive carcinoma; HR = Hormon receptor; HER2 = human epidermal growth factor receptor 2.
